# A Four-Generational Report on Hereditary Head and Neck Paraganglioma

**DOI:** 10.7759/cureus.24143

**Published:** 2022-04-14

**Authors:** Mihnea Cristian Trache, Julian Bewarder, Christian Stephan Betz, Nikolaus Möckelmann, Arne Böttcher

**Affiliations:** 1 Otolaryngology - Head and Neck Surgery, University Medical Center Hamburg-Eppendorf (UKE), Hamburg, DEU; 2 Otolaryngology - Head and Neck Surgery, Kath. Marienkrankenhaus gGmbH, Hamburg, DEU

**Keywords:** sdhd mutation, jugular paraganglioma, carotid body tumor, glomus tumor, head and neck paraganglioma

## Abstract

Background

This article investigates the inheritance, penetrance, clinical presentation, and therapeutic outcomes of hereditary head and neck paragangliomas (HNPGLs) by offering a four-generational report of an 18-member family affected by this rare condition.

Methodology

Information was compiled by examination of patients and a review of medical records and correspondence (retrospective case series).

Results

Six members of the 18-member family were diagnosed with HNPGL between 2002 and 2018. A known pathogenic point mutation in subunit D of the succinyl dehydrogenase complex (SDHD, c.317G>T, p.Gly106Val) was responsible for the tumor phenotype. The mutation could be revealed in seven family members, three diseased adults, one healthy adult, and three healthy children, out of the nine who consented to gene testing. The median age at diagnosis was 33.5 years (range: 22-50 years). Five of the eight primary tumors were glomus caroticum, two were glomus jugulare, and one was a glomus vagale tumor. The therapeutic approaches were multimodal and included embolization therapy, surgery, radiation, and watchful waiting. Follow-up was reported for five of the six patients (mean follow-up of 34.8 months after primary therapy); three showed no disease progression or recurrence.

Conclusions

This study exemplifies the autosomal dominant, parent-of-origin-dependent inheritance and the high disease penetrance in hereditary paraganglioma-pheochromocytoma syndromes. Six out of a total of eight adult descendants (75%) of the original SDHD mutation carrier developed tumors, and the morbidity associated with the disease as well as its therapy was especially high in late-diagnosed, advanced cases. This substantiates the necessity for early radiologic surveillance and genetic testing.

## Introduction

Head and neck paragangliomas (HNPGLs) are neural-crest-derived, usually benign, and hormonally inactive tumors originating from the parasympathetic ganglia in the head and neck region. Typical localizations include the temporal bone and skull base, the soft tissues of the neck, as well as the parapharyngeal space, leading to a variety of clinical manifestations [1].

Up to half of the reported cases occur as hereditary paraganglioma-pheochromocytoma (PGL/PCC) syndromes and follow autosomal dominant inheritance [2]. The gene most frequently affected in hereditary HNPGLs (up to 50%) codes for subunit D of the succinyl dehydrogenase complex (SDHD), a mitochondrial key enzyme of the citrate cycle. Mutations of the other subunits (SDHB,SDHC) are responsible for a minority of cases and show an autosomal dominant inheritance. In total, 12 different genes are associated with hereditary HNPGLs [2]. In SDHD mutations, the disease is generally transmitted on a paternal-only basis and maternal transmission almost never penetrates phenotypically, which is consistent with maternal imprinting (inactivation) of the SDHD locus. However, evidence of corresponding epigenetic changes such as DNA methylations is missing. Furthermore, the SDHD gene shows biallelic expression in other organs such as the brain, kidney, and lymph nodes [3], challenging the imprinting theory. Fluorescence in situ hybridization (FISH) experiments on tumor tissue suggest that an exclusive, somatic loss of the maternal chromosome 11 in SDHD-linked paragangliomas might be responsible for tumor formation and parent-of-origin-dependent inheritance [4].

Hereditary paragangliomas provide the first model to connect heritable mitochondrial defects to neoplasia [5]. Mechanistically, the SDHD protein helps anchor the SDH enzyme in the mitochondrial membrane. As part of the citric acid cycle, SDH converts succinate into fumarate. Succinate is an oxygen sensor that stimulates cells to grow under hypoxia and stabilizes a protein called hypoxia-inducible factor 1 alpha (HIF1-a) by preventing its degradation. The overaccumulation of succinate following SDHD lack of function may lead to an upregulation of HIF1-a and its targets, promoting cell growth and angiogenesis and eventually leading to tumor formation [6]. However, the fundamental aspects of PGL pathogenesis, including the mechanism of reactive oxygen species (ROS) accumulation and the precise role of SDH in the regulation of oxygen homeostasis, are still under inquiry.

Although the exact pathophysiology leading to the individual phenotypes of hereditary HNPGLs is yet to be fully understood, there is empirical data available to determine the age-dependent penetrance as well as the risk for malignancy given a pathological germline mutation. Both these essential parameters depend greatly on the mutated gene. The chance of neoplasia formation by 30 years of age is estimated at 48% in SDHD and only 29% in SDHB mutation carriers according to population-based genetic screening studies [7]. Conversely, the risk for malignant tumors is low (4%) with the more prevalent SDHD mutations but disquietingly high (41%) in the case of SDHB, further underlining the necessity for early surveillance in case of a positive genotype. Reports from individual families over more generations can help further clarify parent-of-origin effects and age-dependent penetrance of this rare condition and better inform the diagnostic and therapeutic process.

## Materials and methods

Study design

This is a retrospective case series. Genealogy investigation and pedigree reconstruction of an 18-member family are presented. For the six affected patients, the clinical manifestations and therapeutic approaches are also described.

Inclusion and exclusion criteria

We included all family members of the index patient (3/III) going back two generations and forward one generation.

Data collection

Information was compiled by examination of patients and a review of medical records and correspondence.

Statistical analysis

Common descriptive statistics (such as percentage, mean, median, and range) were used to summarize the data.

## Results

**Figure 1:**
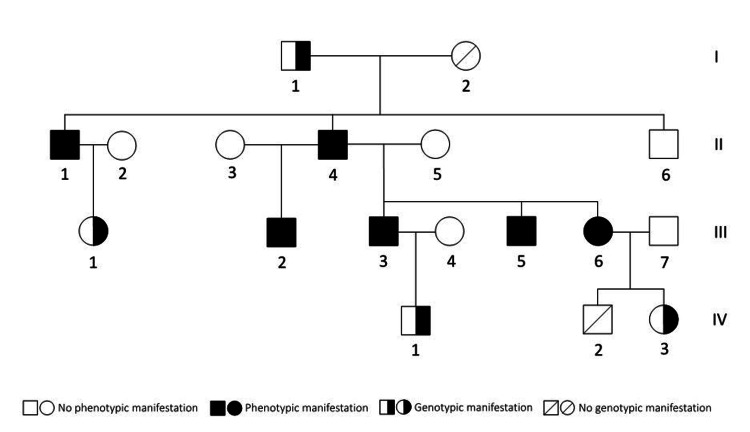
Genealogy tree of the affected family. Circle: female; square: male. Phenotypes: 1/II, 4/II, 2/III: unilateral glomus jugulare tumor; 5/III: bilateral glomus jugulare tumor; 6/III: unilateral glomus vagale tumor; 3/III: bilateral glomus jugulare tumor

**Figure 2:**
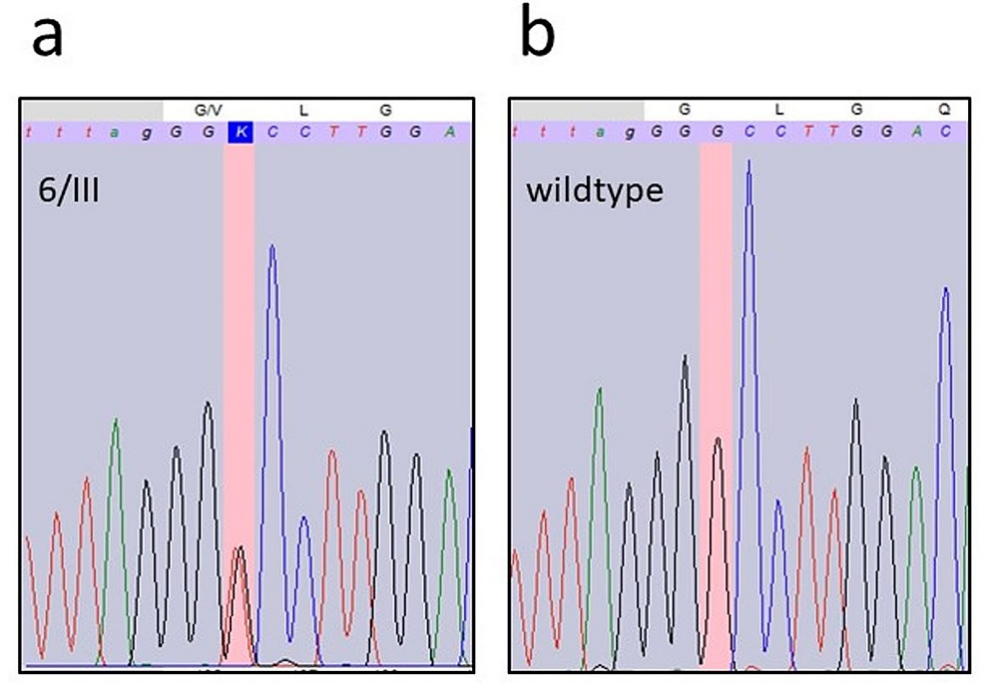
DNA sequencing results. (a) DNA chromatogram of the SDHD gene in patient 6/III, showing a heterozygous point mutation from guanine to thymine at the cDNA-position 317 (c.317G>T). (b) DNA chromatogram of the wildtype SDHD gene. Nucleobases (on highlighted background): A, a, green = adenine; C, c, blue = cytosine; G, g, black = guanine; T, t, red = thymine; K = keto (T or G). Amino acids (on plain background): Q = glutamine; L = leucine; G = glycine; V = valine

A total of six members (five males and a female) of the 18-member family including spouses were diagnosed with benign HNPGL between 2002 and 2018 (Figure 1). Gene sequencing was initially performed in 2017 (Figure 2). A known pathogenic SDHD mutation (c.317G>T, p.Gly106Val) was revealed in seven family members, three diseased adults, one healthy adult, and three healthy children (Figures 1, 2), out of the nine who consented to gene testing. The mutation was traced back four generations, and all the diseased patients who were tested showed a positive genotype.

**Figure 3:**
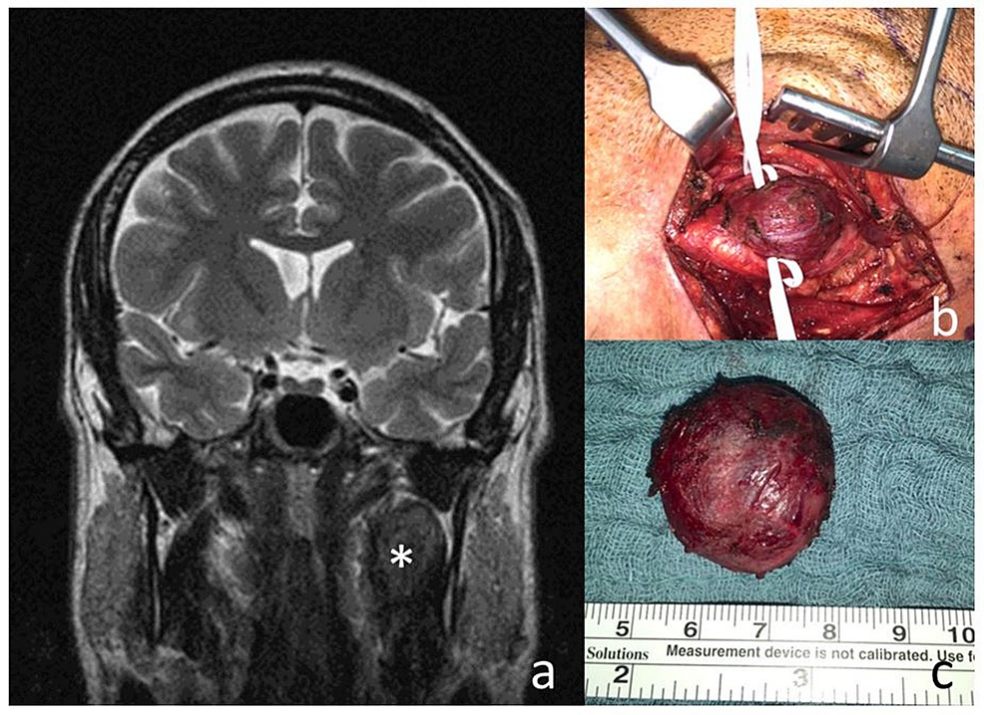
Left-sided glomus caroticum tumor in patient 5/III. (a) Coronal MRI showing the carotid body tumor (*) in the left neck. (b) Intraoperative view with vessel loops around the internal and external carotid artery; right = cranial, up = anterior. (c) In toto resected tumor.

Six out of the eight adult family members (excluding spouses) were diagnosed with HNPGL. Four of them suffered from carotid body tumors (Figure 1 - 1/II, 4/II, 2/III, 5/III), one of which was bilateral (Figure 1 - 5/III, Figure 3), one suffered from bilateral glomus jugulare tumors with intracranial involvement (Figure 1 - 3/III), and one from a unilateral glomus vagale tumor localized medially from the parotid gland (Figure 1 - 6/III). The median age at first diagnosis was 33.5 years (range, 22-50 years). All diagnosed patients were symptomatic at referral.

Symptoms related to the space-occupying effect of the lesion included headache, vertigo, hearing loss, dysphagia, or a visible cervical mass, but no cranial nerve palsy or endocrine symptoms were reported at first referral. The therapeutic approach was multimodal in most of the cases. Three of the five carotid body tumors were addressed by embolization and subsequent resection, one by surgery alone and one by a watch-and-wait strategy. The unilateral glomus vagale tumor was primarily irradiated. Lastly, the bilateral glomus jugulare tumor was primarily managed by embolization (left) and radiotherapy (right).

The patient suffering from the bilateral glomus jugulare tumor was initially referred with a subtotal obstruction of the left external ear canal. The exploration resulted in massive bleeding, which could be stopped by pressure dressing. Further imaging showed bilateral tumors with involvement of the carotid canal wall. The embolization therapy on the left side was complicated in this case by cerebellar and thalamic infarctions, which remained clinically silent but for transitory dysarthria. The left-sided hearing was subsequently lost while the right-sided hearing deteriorated. After two years, the patient suffered a malignant middle cerebral artery infarction on the left side and is now wheelchair-bound.

Follow-up was reported for five of the six patients (mean follow-up of 34.8 months after primary therapy). One patient showed disease progression (5/III), three showed no disease progression after primary therapy, and one showed recurrence after nine months (3/III), which was treated by radiotherapy without further relapse. The SDHD gene mutation was transmitted to two of the three children in the last generation (Figure 1 - 1/IV, 3/IV) and was traced back to the great-grandfather of the family (Figure 1 - 1/I). Interestingly, despite his advanced age, he was the only healthy adult mutation carrier in the family.

## Discussion

The diagnosis of hereditary HNPGL syndrome via gene testing occurred after the detection of bilateral glomus caroticum tumors in one of the family members (Figure 1 - 3/III) 15 years after the first diagnosis of HNPGL in the family. During this time, four prior cases of HNPGL had occurred in the family. In one case, the tumor diameter measured 5 cm at diagnosis (glomus caroticum, Figure 1 - 4/II). Another patient had presented with an unresectable tumor causing subtotal displacement of the auditory canal and suffered from downstream cerebrovascular complications from the embolization therapy (Figure 1 - 5/III). These cases underline the importance of early genetic and radiological screening. At present, multi-gene testing (RET, SDHB, SDHC, SDHD, VGL, SDHAF2, TMEM127, and MAX) is recommended for all patients presenting with HNPGL, and targeted genetic testing is recommended for all adult first-degree relatives of a mutation carrier. Asymptomatic carriers need to be examined clinically and by MRI, skull base to the symphysis pubis, on a twice-yearly basis starting at 16 years of age [8]. The risk of malignant disease in hereditary paraganglioma-pheochromocytoma syndromes is generally low (<5%). There was no malignant disease in our study cohort. Predictive factors for underlying malignancy are pain and a rapidly enlarging neck mass [9]. However, SDHB gene mutations and urinary metanephrine secretion are important risk factors associated with the presence of pheochromocytoma as well as with malignant development [10,11]. Therefore, annual biochemical screening is recommended in addition to MRI monitoring [2]. No biochemical data were available for the patients in this series.

The therapeutic strategy depends on tumor localization, size, growth, and patient factors. Because most HNPGLs are benign, hormonally inactive tumors, the primary therapeutic goal is complete removal. However, the surgical approach is complicated by the inconvenient localization, especially in the case of glomus jugulare tumors, as well as the typically strong vascularization of the tumor. Preoperative embolization can facilitate the resection of carotid body tumors. In the present series, the majority of the carotid body tumors were primarily treated by embolization and subsequent resection. However, a recent meta-analysis showed no significant difference in estimated blood loss, operative time, risk of cranial nerve injury, or stroke between embolization and non-embolization groups of carotid body tumors [12]. Radiotherapy is a further treatment option, especially in glomus jugulare and glomus vagale tumors, particularly in cases where complete surgical resection comes at the cost of lower cranial nerve function. Radiation of glomus jugulare and glomus vagale tumors achieves similar local control rates as with surgery but shows significantly less therapy-associated morbidity [13,14]. Here, radiation therapy was used to treat a glomus vagale (Figure 1 - III/6) and a relapsing glomus caroticum tumor (Figure 1 - 3/III). Due to the slow-growing and generally benign nature of HNPGLs, an active surveillance approach has recently emerged as a suitable alternative, especially for poor surgical candidates or multifocal disease, as was the case in patient 5/III [15].

In our study, the gene variant was traced to the great-grandfather of the family (Figure 1 - 1/I) who was interestingly asymptomatic. Because of the parent-of-origin-dependent penetrance of SDHD mutations, this implies that the mutation had most likely originated with his mother. However, further research is needed to clarify the exact mechanism of parent-of-origin transmission. Large-scale genetic analyses of tumor tissue might be helpful, although they are presently not performed in the clinical routine.

## Conclusions

Hereditary paraganglioma-pheochromocytoma syndromes have an incidence of only approximately 0,6/100.000, making it challenging to investigate their genotype and phenotypic manifestations. This study offers a complete pedigree reconstruction of an 18-member family suffering from this rare disease and summarizes the clinical course of the six affected family members. The responsible point mutation of the SDHD gene has been demonstrated by sequencing and traced back to the great-grandfather of the family. The genealogy tree presented here confirms the autosomal dominant inheritance and the high disease penetrance in hereditary paraganglioma-pheochromocytoma syndromes. Six of the eight adult descendants of the initial mutation carrier developed tumors. The morbidity associated with the tumor as well as the therapy complications were especially high in late-diagnosed, advanced cases. This substantiates the necessity for early radiologic surveillance and genetic testing. Although the pattern of the transmission shown here is in line with the paternal-only inheritance of the tumor phenotype in SDHD mutations, further research is needed to clarify the exact mechanism of this parent-of-origin effect.
